# Investigation of early axonal phenotypes in an iPSC-derived ALS cellular model using a microfluidic device

**DOI:** 10.3389/fncel.2025.1590732

**Published:** 2025-07-24

**Authors:** Asako Otomo, Keiko Nishijima, Yuta Murakami, Mitsuru Ishikawa, Haruka Yudahira, Kento Shimakura, Hideyuki Okano, Masashi Aoki, Hiroshi Kimura, Shinji Hadano

**Affiliations:** ^1^Molecular Neuropathobiology Laboratory, Department of Physiology, Tokai University School of Medicine, Isehara, Kanagawa, Japan; ^2^Micro/Nano Technology Center, Tokai University, Hiratsuka, Kanagawa, Japan; ^3^Department of Mechanical Engineering, Tokai University School of Engineering, Hiratsuka, Kanagawa, Japan; ^4^Division of CNS Regeneration and Drug Discovery, International Center for Brain Science, Fujita Health University, Aichi, Japan; ^5^Department of Physiology, Keio University School of Medicine, Tokyo, Japan; ^6^Department of Neurology, Tohoku University Graduate School of Medicine, Sendai, Japan

**Keywords:** amyotrophic lateral sclerosis (ALS), iPSCs, microfluidic device, FUS/TLS, lower motor neurons

## Abstract

**Introduction:**

Amyotrophic lateral sclerosis (ALS) is a progressive neurodegenerative disease caused by the loss of upper and lower motor neurons. Mutations in the FUS/TLS gene have been reported as the second most common mutation in Japanese patients with familial ALS. In recent years, lower motor neurons (LMNs) differentiated from induced pluripotent stem cells (iPSCs) derived from ALS patients have been widely used to analyze the mechanisms of neuronal cell death and degeneration.

**Methods:**

In this study, we developed a microfluidic device designed to observe axonal growth, morphology, and trafficking at high resolution in neurons derived from induced pluripotent stem cells (iPSCs) and tested whether our microfluidic device effectively evaluates neurodegenerative phenotypes. We used iPSCs carrying homozygous FUS/TLS mutations (FUS_H517D) to induce LMNs by expressing NEUROG2, ISL1, and LHX3 under the control of the tetracycline regulation system.

**Results and discussions:**

After seven days of in vitro differentiation (DIV7), we confirmed that over 95% of iPSCs differentiated into HB9-positive LMNs. Notably, the cell viability of FUS_H517D LMNs was comparable to that of LMNs differentiated from iPSCs without the FUS/TLS mutation at DIV7. However, by DIV14 and DIV21, the viability of FUS_H517D LMNs was notably lower than that of control LMNs, indicating degeneration of FUS_H517D LMNs after differentiation. Using our microfluidic device, we assessed axonal phenotypes in FUS_H517D LMNs. Under oxidative stress conditions, we observed that the axonal length of FUS_H517D LMNs was significantly shorter than that of control cells as early as DIV7, with this axonal growth restriction becoming more pronounced by DIV11. This suggests that axonal growth restriction is an early detectable phenotype in degenerating neurons. Additionally, we examined mitochondrial trafficking within axons in our device, which is often disrupted in degenerative neurons. Our results showed a significant increase in the number of motile mitochondria in FUS_H517D LMNs, with retrograde transport accounting for a large portion of trafficking. Our microfluidic device-based culture and evaluation system using FUS_H517D LMNs offers a valuable ALS cellular model focused on early axonal phenotypes. This approach contributes to the study of molecular mechanisms underlying axonal degeneration in ALS.

## 1 Introduction

Amyotrophic lateral sclerosis (ALS) is a progressive neurodegenerative disease characterized by the degeneration of motor neurons in the motor cortex, brainstem, and spinal cord. Approximately 10% of ALS cases are familial, with over 40 causative genes identified (Akçimen et al., 2023). Mutations in the *FUS/TLS* gene account for about 4% of familial ALS cases worldwide and 10% in Japan (Suzuki et al., 2023; Akçimen et al., 2023). Symptoms of *FUS/TLS*-linked ALS (ALS6) typically begin in the fourth decade of life and often progress rapidly (Suzuki et al., 2010; Hübers et al., 2015). Mutations in the *FUS/TLS* gene lead to mislocalization of the FUS protein (FUS) from the nucleus to the cytoplasm in both postmortem brain and spinal cord tissue of patients, as well as in patient-derived cells (Kwiatkowski et al., 2009; Vance et al., 2009). Mutant FUS can aggregate in the cytoplasm, resulting in neuronal dysfunction (Kwiatkowski et al., 2009; Vance et al., 2009). FUS is a DNA/RNA binding protein structurally similar to TAR DNA binding protein 43 (TDP-43), suggesting that FUS may share similar functions with TDP-43. TDP-43, a protein encoded by the ALS-causing gene *TARDBP*, is involved in RNA splicing, metabolism, and trafficking (Mackenzie et al., 2010; Ishiguro and Ishihama, 2022). Consequently, alterations in RNA splicing, trafficking, and the regulation of gene expression may play a role in ALS pathogenesis. However, the molecular pathogenesis underlying ALS6 remains incompletely understood.

In recent years, lower motor neurons (LMNs) derived from iPSCs established from ALS patients have become a valuable tool for analyzing neuronal cellular pathology and for drug discovery related to ALS (Egawa et al., 2012; Farkhondeh et al., 2019; Fujimori et al., 2018). These LMNs, differentiated from ALS patient-derived iPSCs, can replicate the cellular pathology observed in ALS patients’ central nervous systems *in vitro*, making them an ideal cellular model for studying ALS pathogenesis. LMNs can be generated from embryonic stem cells (ESCs) and iPSCs by mimicking extracellular developmental signaling cues (Chambers et al., 2009; Surmacz et al., 2012). However, this process is time-consuming and involves multiple steps, including treatment with inhibitors or activators of signaling molecules, which makes it difficult to control experimental conditions and can lead to batch effects in differentiation efficiency. To address these challenges, we focused on a direct differentiation method for LMNs from iPSCs, achieved by the expression of NEUROG2, ISL1, and LHX3 under tetracycline regulation. The expression of these fate-determining transcription factors enables stem cells to differentiate into cholinergic neurons that closely resemble LMNs (Fernandopulle et al., 2018). These rapidly induced LMNs (Tet-on LMNs) carrying genetic mutations associated with ALS have the potential to serve as a reliable cellular model for ALS, suitable for drug screening and development, as they allow for the production of a large number of neurons with consistent quality.

Neurons are highly polarized cells, typically consisting of a single long axon and multiple dendrites. Each part of the neuron has distinct functions, so to understand these compartment-specific functions, it is essential to observe and analyze phenomena within each specific compartment. However, using conventional *in vitro* cell culture methods, it is challenging to focus on particular compartments of neurons, as axons and dendrites tend to extend randomly and connect to other cells. Various microfluidic devices have been developed to address this challenge, which enables compartment-specific analysis within neurons (Gupta et al., 2022; Neto et al., 2016). They allow researchers to observe specific neuronal compartments and isolate compartment-specific samples. Additionally, these devices help create a microenvironment that more closely mimics *in vivo* conditions by offering controlled 3D structures, extracellular matrix supplementation, and the possibility of co-culture with other cell types. Microfluidic devices typically consist of two compartments separated by long, thin micro-slits, a design widely used in neuroscience research (Taylor et al., 2003; Taylor et al., 2005), and have become commercially available. This compartmentalized structure enables the culture of neurons while keeping cell bodies and axons in separate device compartments. The micro-slits serve as guidance channels, directing axon growth straight toward the axonal compartment while preventing cell bodies from migrating into the axonal compartment. However, when we differentiated iPSC directly to neurons to induce Tet-on LMNs using a commercially available compartmentalized microfluidic device to control the polarization of neurons, we found that the cells migrated into the micro-slits and reached the other compartment during differentiation because the micro-slits’ size was unsuitable for iPSCs. Since the size of the cell body varies among species and changes from small to larger during neuronal development, optimizing the size and structure of micro-slits is required to use the Tet-on LMNs system.

In this study, we developed a microfluidic device suitable for culturing LMNs from human iPSCs. The device was established based on the compartmentalized device design with the structural modification of micro-slits, which enables us to control neuronal polarization and efficiently observe neurons in living and fixed conditions. In addition, we obtained Tet-on NEUROG2/ISL1/LHX3 iPS cell lines with FUS disease-causing mutation (FUS_H517D) and examined whether Tet-on FUS_H517D LMNs show the cellular phenotypes related to ALS. Moreover, using these cell lines, we verified whether a microfluidic device-based assay can uncover axonal phenotypes. Taken together, we propose that our microfluidic device-based cell culture and evaluation system using Tet-on FUS_H517D LMNs might become a helpful tool for ALS drug discovery and evaluation and for understanding the pathogenesis underlying LMN degeneration, focusing on axonal phenotypes.

## 2 Materials and methods

### 2.1 Microfluidic device fabrication

The microfluidic device was fabricated by traditional photolithography and soft lithography (Kimura et al., 2008). The photocurable resin was spin-coated to construct a master mold. By blocking light with a mask, UV irradiation was applied only to the microstructure portion. Remove parts other than the microstructure part with a developing solution. Pour Polydimethylsiloxane (PDMS) into the mold. After curing PDMS, remove the PDMS chip, cut out unnecessary parts, mold the reservoir part, and then bond it with the cover glass. The device was washed with 70% EtOH and dried with UV irradiation. After degassing, the coating solution containing 0.1 mg/mL Poly-D-Lysine (PDL) was introduced into each compartment and incubated for 2 h. The device was washed with distilled water three times and coated with 20 μg/mL Laminin for 2 h.

### 2.2 iPSC culture and iPSC cloning

iPSC (201B7) from RIKEN BRC and FUS iPSCs used in this study were established previously (Ichiyanagi et al., 2016). iPSCs are cultured using StemFit AK02N (Ajinomoto) on an iMatrix 551 coated dish. To obtain Tet-on NEUROG2/ISL1/LHX3 iPSCs, plasmid vector encoded PiggyBac transposase along with PiggyBac Transposon vectors were transfected to iPSCs using GeneJuice Transfection Reagent (Merck Millipore). Transposon vectors encode rtTA or NEUROG2, ISL1, and LHX3 are independently constructed and transfected. Transfected cells were selected by 150μg/mL of Hygromycin and 400 μg/mL of G418 treatment. Several clones were isolated from each cell line and used for experiments.

### 2.3 Differentiation of Tet-on LMNs

Isolated Tet-on NEUROG2/ISL1/LHX3 iPSC clones were dissociated into single cells and plated on 0.01 mg/mL PDL, 20 μg/mL Laminin coated plate or cover glass. They were maintained in the neural differentiation media: NDM [Neurobasal plus system with 100 μM ascorbic acid, 100 μM cAMP, 5 μM DAPT, and 1 μg/mL doxycycline] with 10 μM Y-27632. Twenty four hours after plating the cells, the NDM with 10 μM Y-27632 was removed and replaced with fresh NDM. Half the volume of NDM was replaced with fresh NDM once every 2 or 3 days. Illustrations describing the procedure for 2.1 and 2.2 were included in Supplementary Figure 1.

### 2.4 Antibodies

Antibodies used in this research are listed in Supplementary Table 1.

### 2.5 Immunocytochemistry

Cells were fixed with 4%PFA/PBS (−) for 30min at room temperature. After washing with PBS (−) three times, cells were blocked with a blocking solution [5%NGS, 0.1% Triton-100 in PBS (−)] for 30 min at room temperature. The cells were incubated with primary antibodies diluted in antibody diluting solution [2%NGS, 0.05% Triton-100 in PBS (−)] overnight at 4 degrees. The cells were washed three times with PBS (−) and were incubated with Alexa594 Fluor labeled anti-Rabbit IgG antibody with or without Alexa488 Fluor labeled anti-Mouse IgG diluted in antibody diluting solution overnight at 4 degrees. The cells were washed three times with PBS (−), then stained with Alexa488 Fluor-labeled Phalloidin and DAPI when required. Immunofluorescent signals were visualized and analyzed by BZ X-710 (Keyence) or FV3000 (EVIDENT, OLYMPUS).

### 2.6 Cell viability assay

Cell viability was measured using PrestoBlue reagent (ThermoFisher Scientific), and the result was shown as a ratio of cell viability at DIV14 and DIV21 to that at DIV7. Experiments were repeated three times, and representative results were shown.

### 2.7 Quantification of HB9-positive cells

After 4% PFA fixation, we stained the cells using specific primary antibodies for NF200 and HB9. Primary antibodies were visualized by the Alexa fluorescence-labeled secondary antibodies. Nucleus were stained with DAPI. Fluorescent signals were visualized and analyzed by Keyence BZ X-710 using a dynamic cell count module. HB9-positive cells among DAPI-stained cells were counted, and the percentage of HB9-positive cells in images was calculated. Four areas (1mm^2^) in the sample were independently analyzed. The sample was duplicated at an experiment, and the experiments were conducted twice. The number of cells used for this analysis: Control_4; *n* = 1,709, Control_9; *n* = 1,497, FUS_1_1; *n* = 741, FUS_1_4; *n* = 1,259, FUS_2_3; *n* = 629.

### 2.8 Quantitative PCR

Total RNAs from Control_4 and FUS_H517D Tet-on LMN at DIV14 were purified using an RNeasy mini kit (QIAGEN). A purified 20 ng of total RNAs was used for a reaction of One Step TB Green PrimeScript PLUS RT-PCR (TAKARA bio Inc.) by StepOneplus real-time PCR system (Appliedbiosystem). The results were analyzed using Design and Analysis software 2.8 (Appliedbiosystem). Primers used for this experiment are listed in Supplementary Table 2.

### 2.9 Differentiation of Tet-on LMNs in the device

iPSCs were treated with TrypLE Select Enzyme diluted into 1:1 with PBS (−) containing 200μM EDTA and dissociated into a single cell in NDM. 200000 cells were introduced into the cell culture compartment of the device. Half the volume of NDM was changed once every 2 days. When we treated the cells with NDM without antioxidants, we changed NDM containing 1XB27 supplement without antioxidants (ThermoFisher Scientific, 10889038) instead of 1XB27 plus supplement at 3 days of culture.

### 2.10 Sholl analysis

Sholl analysis was conducted using the Sholl tool in the neuroanatomy Plugin for Image-J2(Fiji). The gap between lines was set at 35 μm. Cells were stained with TUBB3 antibody to visualize the neurite structures, and each cell was pictured by Keyence BZ X-710. Images were binarized and used for analysis. The number of intersections passing a line was counted, and the average number of the intersections was shown in the graph. More than 50 cells were analyzed for each condition. Representative binary images showing whole cell morphology used in Sholl analysis are shown in Supplementary Figure 3.

### 2.11 ROS detection

Differentiated Tet-on LMNs at DIV11 were changed to media with or without antioxidant (AO) containing 2.5 μM CellROX green reagent for 24 h. The cells were washed twice with PBS (−) and fixed with 4% PFA. The samples were mounted with mounting media with DAPI (Vector Laboratories). Fluorescent signals were visualized and analyzed by Keyence BZ X-710 using a dynamic cell count module. CellROX green-positive cells among DAPI-stained nuclei were counted. Then, the ratio of CellROX green-positive cells in images was calculated. More than 10 images from two independent samples were analyzed. The number of cells used for this analysis: Control_4 conventional; *n* = 205, Control_4−AO; *n* = 224, FUS_1_4 conventional; *n* = 220, FUS_1_4−AO; *n* = 255.

### 2.12 Quantification of axonal length and number

NF 200 positive axons that passed through the micro-slits in the axonal compartment were traced by Neuron-J in Image-J2 (Fiji), and the length of axons traced in the axonal compartment at DIV7, DIV11, and DIV14 was calculated using one microfluidic chip including two axonal compartments. Experiments were conducted three times for DIV7 and DIV11, and once for DIV14 and DIV16, and representative data were shown. The number of axons detected in a single axonal compartment was counted and shown in the graph (Figure 7E). The number of axonal compartments analyzed in this analysis is as follows: Control_4 DIV7 (*n* = 6), FUS_1_4 (*n* = 6), Control_4 DIV11 (*n* = 5), FUS_1_4 DIV11 (*n* = 5), Control_4 DIV14 and 16 (*n* = 3), FUS_1_4 DIV14 and 16 (*n* = 3).

**FIGURE 1:**
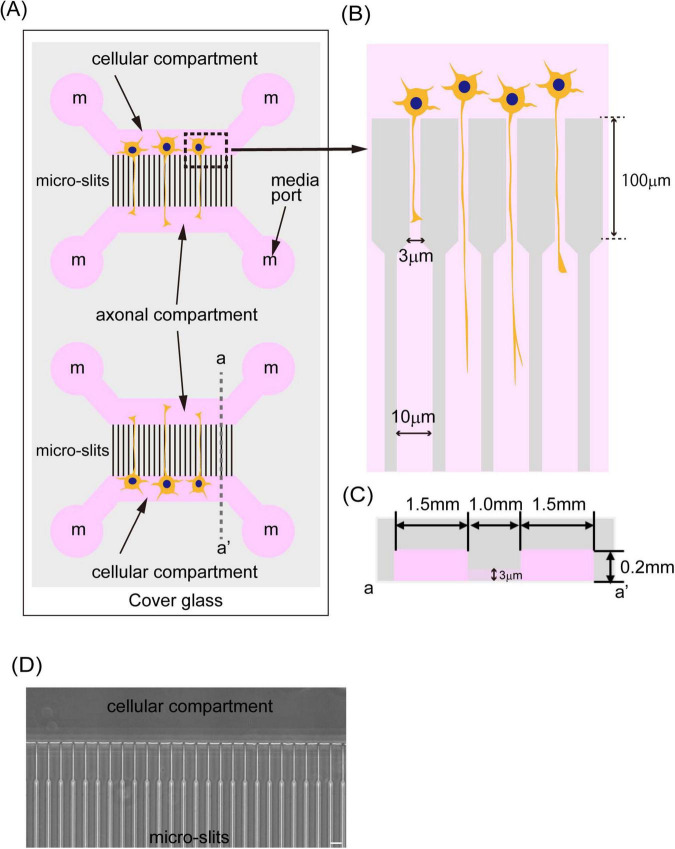
A schematic image of the microfluidic device. **(A)** The illustration shows a schematic image of the microfluidic device. The device was fabricated by PDMS and bound to cover glass around 0.17 mm thickness. The PDMS chip has two independent culture areas with four independent media ports (media ports were indicated and labeled with “m”). Two of them supply media to the cellular compartment where cells are cultivated, and the other two media ports supply media to the axonal compartment where axons elongate. Those two compartments were connected with 1,000 μm long micro-slits. **(B)** The illustration shows the structure of micro-slits in the device. Micro-slits facing the cells were designed to be 3 μm wide, 4 μm high, and have a 100 μm long fine structure to avoid cell migration. **(C)** The illustration shows a schematic image of a cross-section of the device indicated by dash line a-a’ in **(A)**. Cellular and axonal compartments have a 1.5 mm length, are 4 mm wide, and are 0.2 mm high. **(D)** The phase contrast image of micro-slits in the device was shown. The scale bar indicates 20 μm.

**FIGURE 2:**
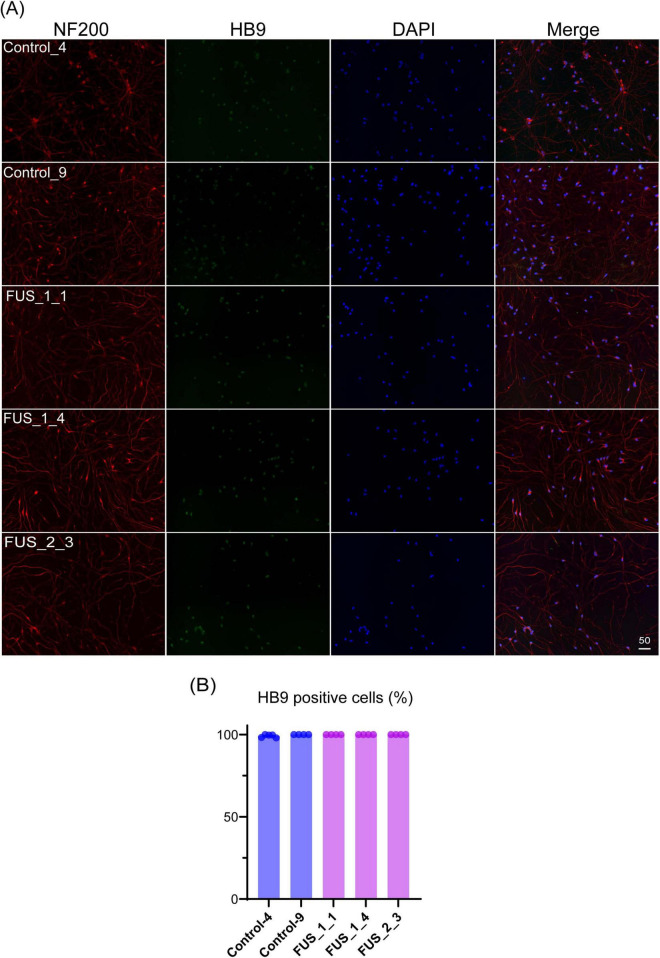
Tet-on NEUROG2/ISL1/LHX3 iPSCs express LMN markers. **(A)** Immunofluorescent co-staining of HB9 (green) and NF200 (red) on the culture at DIV7. Nuclei were stained by DAPI (blue). The scale bars indicate 50 μm. Control_4 and Control_9 were established from control iPSC, and FUS_1_1, FUS_1_4, and FUS_2_3 were established from iPSC clones carrying homozygous FUS_H517D mutations. **(B)** HB9-positive cells among DAPI-stained cells were counted, and the percentage of HB9-positive cells in images is shown in the graph. Experiments were conducted independently twice. The number of cells used for this analysis: Control_4; *n* = 1,709, Control_9; *n* = 1,497, FUS_1_1; *n* = 741, FUS_1_4; *n* = 1259, FUS_2_3; *n* = 629.

**FIGURE 3:**
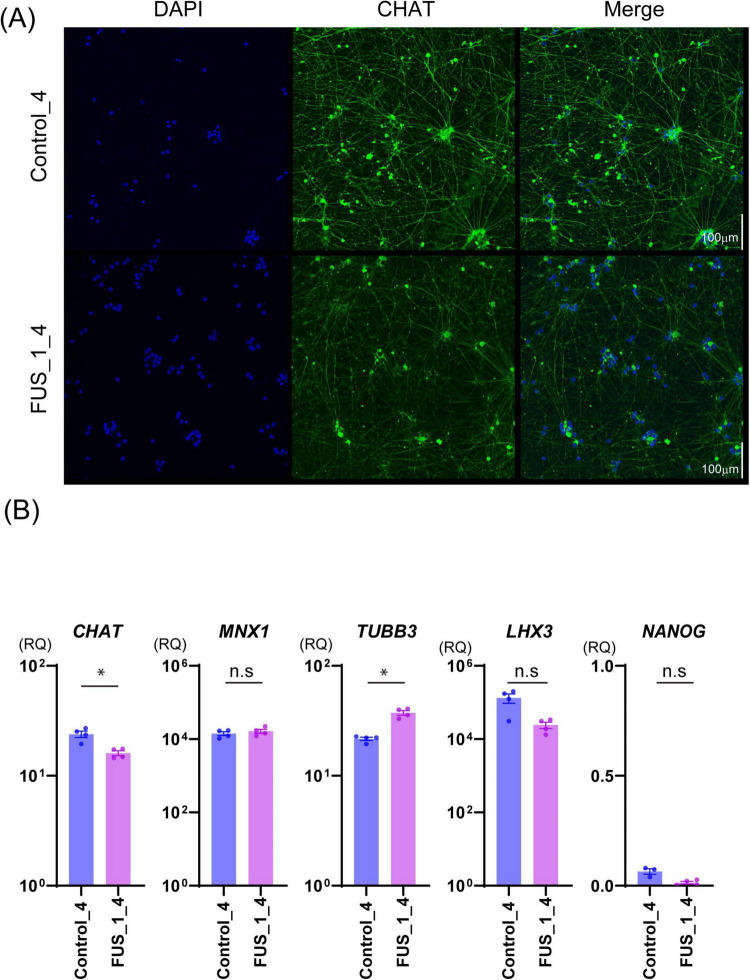
FUS_H517D Tet-on LMNs express *CHAT* and *MNX1(HB9)*. **(A)** Immunofluorescent staining of CHAT (green) on the culture at DIV35 in Control_4 and FUS_1_4. Nuclei were stained by DAPI (blue). Z-stack images were captured by confocal microscopy and presented as maximum projection images. The scale bar indicates 100 μm. **(B)** The relative quantities of *CHAT*, *MNX1*, *TUBB3*, *LHX3*, and *NANOG* expressions in Control_4 and FUS_1_4 LMNs at DIV 14 are shown. The relative quantities were calculated based on the marker’s expression levels in iPSCs before differentiation. Experiments were conducted independently three times. A representative series of results is shown. The statistical difference between groups was assessed by the Mann-Whitney Test. *p < 0.05.

**FIGURE 4:**
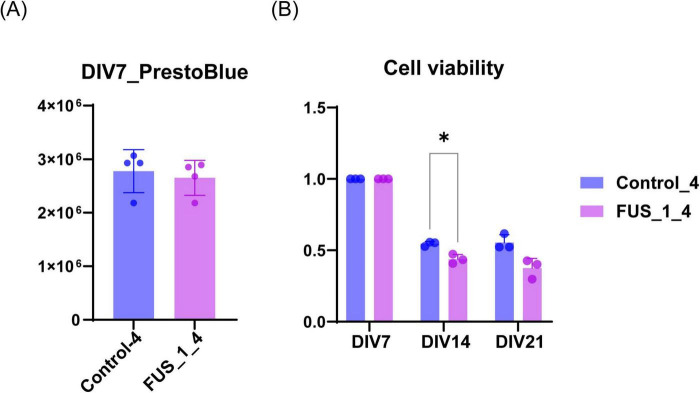
FUS_H517D Tet-on LMNs represent lower cell viability. **(A)** The cell viability of FUS_H517D Tet-on LMNs and control LMNs at DIV7 were analyzed using PrestBlue reagent, and the results are shown by fluorescent intensities. **(B)** Cell viabilities of FUS_H517D Tet-on LMNs and control LMNs at DIV14 and DIV21 were shown. The results are shown as a ratio of cell viability at DIV14 and DIV21 to that at DIV7. Experiments were repeated three times, and representative results are shown. The difference between the groups was assessed using a two-way ANOVA followed by a Bonferroni post hoc test.*p < 0.05.

**FIGURE 5:**
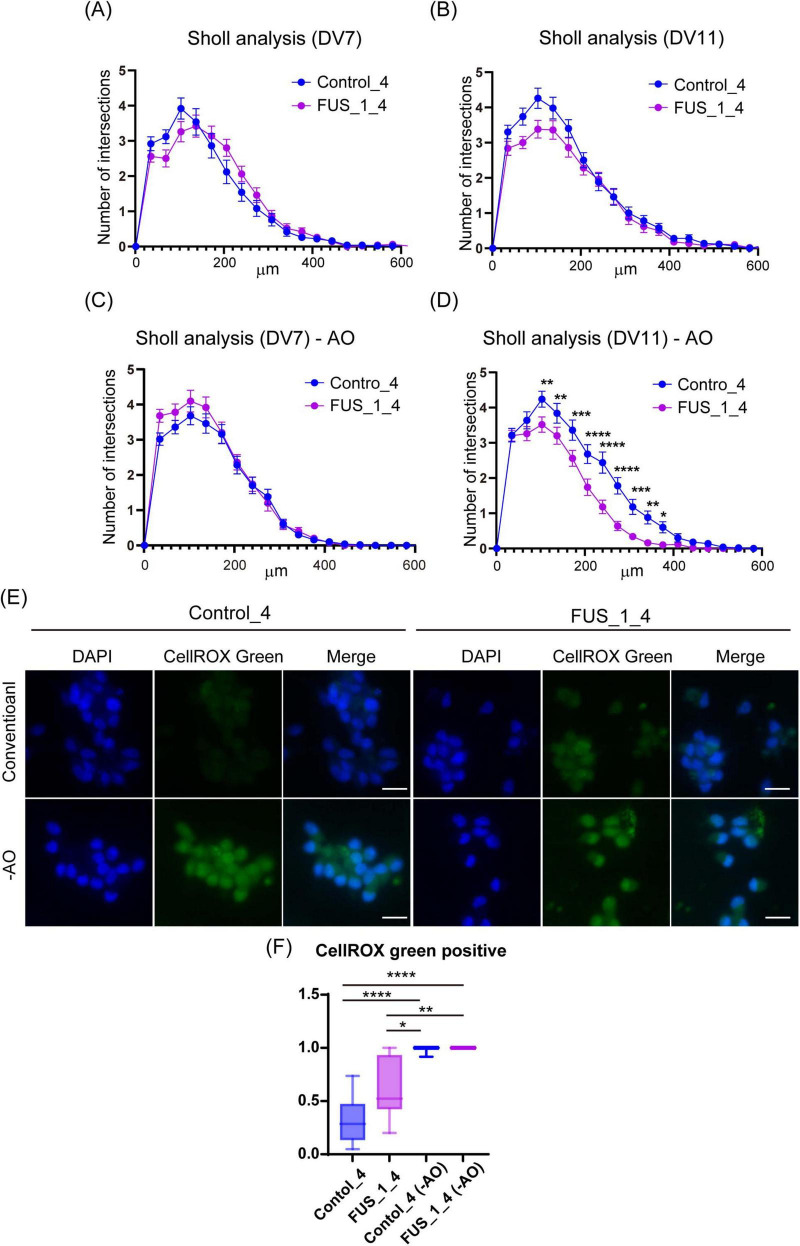
FUS_H517D Tet-on LMNs represent lower neurite complexity at DIV11 but not DIV7 under oxidative stress. **(A)** The results of the Sholl analysis at DIV7 are shown. The horizontal axis indicates the distance from the center of the cell, whereas the vertical axis indicates the average number of cross points on concentric circles passed through by neurites. **(B)** The results of the Sholl analysis at DIV11 are shown. **(C)** The results of the Sholl analysis at DIV7 under oxidative stress are shown. **(D)** The results of the Sholl analysis at DIV11 under oxidative stress are shown. Each group used over 50 cells to analyze neurite morphology. The difference between the groups was assessed by *two-way ANOVA* followed by *Tukey’s multiple comparisons test.* *p < 0.05, **p < 0.01, ***p < 0.001, ****p < 0.0001. **(E)** CellROX Green staining of conventional and oxidative stress conditions. ROS were detected using CellROX Green (Thermo Fisher Scientific), a fluorescent dye that binds to DNA and emits bright green fluorescence upon oxidation by ROS. Cells were cultured for 24 h in antioxidant-free (−AO) media, after which ROS levels were assessed at DIV12. **(F)** The Ratio of CellROX green-positive cells in the image was shown. More than 10 images from two independent samples were analyzed. (The number of cells used for this analysis: Control_4 conventional; *n* = 205, Control_4−AO; *n* = 224, FUS_1_4 conventional; *n* = 220, FUS_1_4−AO; *n* = 255). The difference among medians was assessed by the Kruskal-Wallis Test and statistical significance was tested by Dunn’s multiple comparisons test. *p < 0.05, **p < 0.01, ****p < 0.0001.

**FIGURE 6:**
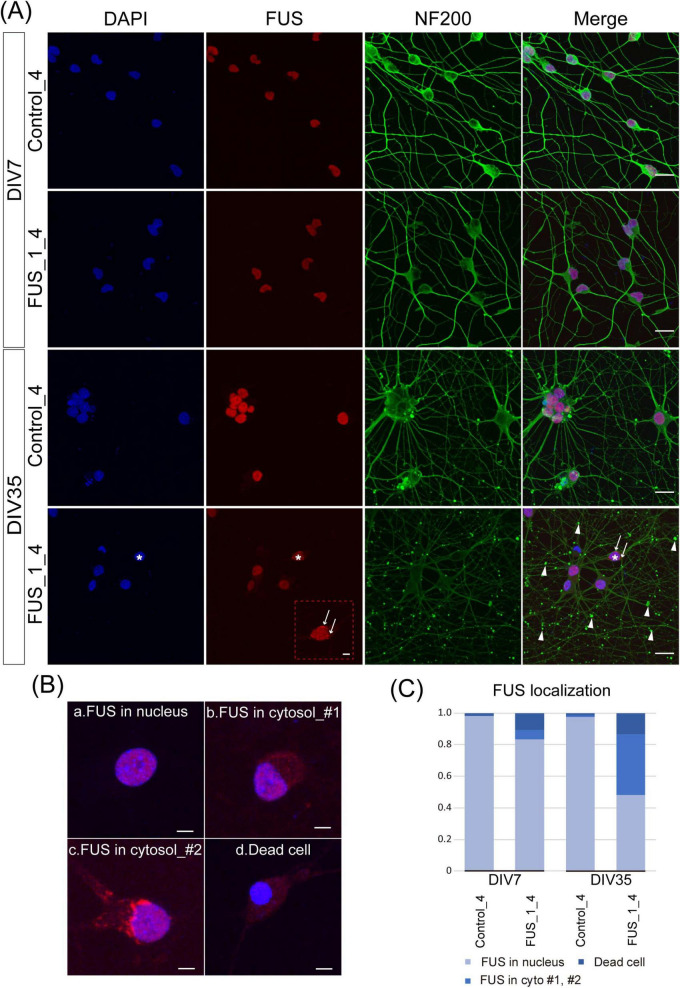
Extranuclear FUS was detected in FUS_H517D Tet-on LMNs. **(A)** Immunofluorescent co-staining of NF200 (green) and FUS (red) in Control_4 and FUS_1_4 LMNs at DIV7 and DIV35. Nuclei were stained by DAPI (blue). The scale bars indicate 20 μm. At DIV7, both Control_4 and FUS_1_4 LMNs, FUS localization was mainly detected in the nucleus. On the other hand, extranuclear localization of FUS was detectable in FUS_1_4 LMNs at DIV35, as indicated by white arrows in the enlarged image of FUS staining (The scale bar indicates 5 μm). White arrows and arrowheads indicate FUS aggregation and swollen axons, respectively. *FUS_1_4 DIV35 DAPI (blue) and FUS (red) staining are used to indicate the cell shown enlarged in FUS (red) staining. **(B)** Representative images of FUS localization. (a) nuclear localization of FUS, (b) extranuclear (cytosolic) localization of FUS_#1, (c) extranuclear (cytosolic) localization of FUS_#2 (with aggregation), and (d) condensed nuclei lacking detectable FUS localization. The scale bars indicate 5 μm. **(C)** Quantification of the FUS localization. FUS localization was categorized into three populations based on the example shown in **(B)**. The ratio of the FUS cytoplasmic localization was dramatically increased in FUS_1_4 at DIV35. Quantification was performed using 5–6 independent confocal images from two independent samples at each time point (The number of cells used for this analysis: Control_4 DIV7: *n* = 168, DIV35: *n* = 139, FUS_1_4 DIV7: *n* = 173, DIV35: *n* = 202). The difference in frequencies among groups was analyzed using a χ^2^ test, which revealed a statistically significant result (*p* < 0.001).

**FIGURE 7:**
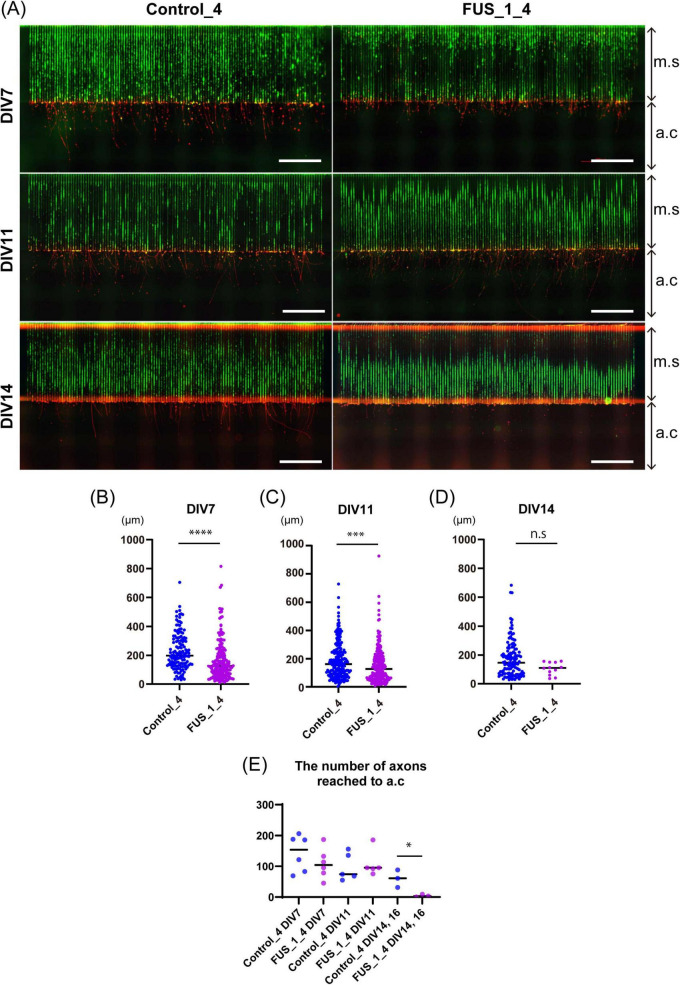
Axonal growth of FUS_H517D Tet-on LMNs was suppressed in oxidative stress. **(A)** Representative immunofluorescent co-staining of Alexa488 Phalloidin (green) and NF200 (red) on Control_4 and FUS_H517D Tet-on LMNs in the device at DIV7, DIV11, and DIV14 are shown. The Scale bars indicate 100 μm. m.c and a.c stand for micro-slits or axonal compartments, respectively. **(B)** The length of the axon at DIV7 under oxidative stress. The length of the axon extending from the exit of the micro-slits to the tip of the axon within the axon compartment was traced and measured. Median; Control_4, 198.8 μm (*n* = 138 from one chip, two independent a.c) and FUS_H517D, 127.3 μm (*n* = 180 in one chip, including two independent a.c). The difference between medians was assessed by the Mann-Whitney Test. ****p < 0.0001. **(C)** The axon length elongates in the axonal compartment at DIV11 under oxidative stress. Median; Control_4, 162.5 μm (*n* = 204 in one chip, including two independent a.c) and FUS_H517D, 126.0 μm (*n* = 226 in one chip, including two independent a.c). The difference between medians was assessed by the Mann-Whitney Test. ***p < 0.001. **(D)** The length of the axon elongates in the axonal compartment at DIV14 under oxidative stress. Median; Control_4, 147.3 μm (*n* = 119 in one chip, including two independent a.c) and FUS_H517D, 109.9 μm (*n* = 14 in one chip, including two independent a.c). The difference between medians was assessed by the Mann-Whitney Test. Experiments are conducted three times for DIV7 and DIV11, and once for DIV14 and DIV16, and representative data are shown. **(E)** The number of axons detected in a single a.c. is shown. The number of axons reached a.c at each DIV was compared by a student’s *t*-test. *p < 0.05.

### 2.13 Real-time observation of mitochondria trafficking

Cells in the device were stained with MitoTracker green for 24 h in a CO_2_ incubator. Movies were captured at a micro-slit 500 μm away from the cellular compartment for 23.3 min (280 frames, 5 s/frame) in the incubation chamber of Keyence BZ X-710. Kymographs were generated as previously reported (Otomo et al., 2022) and analyzed using the KymoToolBox Plugin in FIJI. Experiments were conducted three times at DIV12-14 in each sample. More than 200 ROIs from 20 to 21 independent movies were analyzed independently for each cell line.

### 2.14 Statistical analysis

Statistical analysis was conducted by using GraphPad Prism8.

## 3 Results

### 3.1 Development of a microfluidic device suitable for culturing LMNs from human iPSCs

We developed a microfluidic device optimized for culturing LMNs derived from human iPSCs. This device features two distinct compartments—a cellular compartment and an axonal compartment—separated by finely engineered micro-slits (Figure 1A), based on the design originally reported by Taylor (Taylor et al., 2003). The original device, fabricated using polydimethylsiloxane (PDMS), employed 10 μm-wide and 3 μm-high micro-slits to regulate neuronal polarity, and was primarily intended for culturing rodent neurons. We have also reported the advantages of using this type of device with primary cultured neurons from mice (Otomo et al., 2022). More recently, a commercially available platform known as the XonaChip^1^ has been introduced and adopted by various researchers (Linares et al., 2023; Dinamarca et al., 2024). The XonaChip retains a similar design to Taylor’s device but offers improved culture conditions, enabling prolonged culture durations. This makes it particularly suitable for human neurons, which typically require extended periods for differentiation and maturation. However, previous studies have reported that a subset of neurons can migrate into the micro-slits of the XonaChip (Lotlikar et al., 2022).

In our study, we seeded iPSCs into the cellular compartment of the device to initiate differentiation by the expression of transcription factors (Supplementary Figure 1). Given that iPSCs are smaller in size than certain differentiated neurons, we anticipated that the XonaChip’s standard slit dimensions might not effectively prevent cell migration into the axonal compartment. If iPSCs occasionally migrate into and differentiate within the micro-slits, it becomes challenging to identify axons within this region reliably. This can potentially compromise the accuracy of axonal trafficking and morphological analyses. To address this, we modified the micro-slit design in our device. Specifically, the micro-slits adjacent to the cellular compartment were reduced to 3 μm in width, 4 μm in height, and 0.1 mm in length—dimensions intentionally chosen to be smaller than the iPSC cell body, thereby minimizing undesired migration. These micro-slits connect to 10 μm-wide, 0.9 mm-long observation-friendly slits (Figures 1A,B). Each compartment measures 1.5 mm in length, 4 mm in width, and 0.2 mm in height (Figure 1C), providing sufficient space to culture up to 200,000 iPSCs. The total length of the micro-slit region is 1 mm (Figure 1C), which allows only axons, but not dendrites or cell bodies, to access the axonal compartment. The device is fabricated using PDMS and bonded to a glass coverslip, facilitating high-resolution imaging of cellular processes within the micro-slits (Figure 1D).

To establish an ALS cellular model, we obtained Tet-on NEUROG2/ISL1/LHX3 iPSCs with TALLEN-mediated FUS_H517D mutations (Ichiyanagi et al., 2016) and treated them with doxycycline to induce differentiation (Supplementary Figure 1). For controls, we used Tet-on NEUROG2/ISL1/LHX3 iPSCs from the same individual without genetic modifications. After 7 days of differentiation (DIV7), we fixed the cells and stained them with antibodies for HB9 (a lower motor neuron marker) (Arber et al., 1999) and NF200 (a marker for mature neurons). Immunocytochemical staining revealed that almost all the Tet-on NEUROG2/ISL1/LHX3 iPSC clones in this study were double-positive for HB9 and NF200 (Figure 2A). Quantification of HB9-positive cells among DAPI-stained cells showed that over 95% of LMNs in each iPSC line expressed HB9 at DIV7 (Figure 2B). When LHX3, one of the transcription factors induced through the Tet-on system, was highly expressed (Figure 3B), the upregulation of Tubulin Beta 3 (*TUBB3*) and the downregulation of Nanog homeobox (*NANOG*) were also observed, suggesting that iPSCs were differentiated into neurons depending on the Tet-on system. Additionally, we confirmed the expression of choline acetyltransferase (*CHAT*) and HB9 (*MNX1*) in Tet-on LMNs using immunocytostaining (Figures 2A, 3A) and real-time PCR analysis (Figure 3B). These results indicate that the Tet-on LMNs exhibit characteristics consistent with lower motor neurons.

### 3.2 FUS_H517D Tet-on LMNs show lower viability and susceptibility to oxidative stress

Characteristics of iPSC-derived LMNs carrying TALLEN-mediated FUS_H517D mutations (FUS_H517D) have been analyzed and reported (Ichiyanagi et al., 2016). FUS_H517D LMNs have demonstrated lower cell viability, accelerated neurite degeneration, and cytoplasmic mislocalization of the FUS protein (Ichiyanagi et al., 2016). Since LMNs in the previous study were differentiated from iPSCs using conventional methods, we needed to confirm whether FUS_H517D Tet-on LMNs display identical cellular phenotypes.

First, we compared cell viability between FUS_H517D Tet-on LMNs and control Tet-on LMNs. While cell viability was similar for both FUS_H517D and control Tet-on LMNs at DIV7, FUS_H517D Tet-on LMNs showed significantly lower viability than controls at DIV14 and DIV21 (Figures 4A,B). To confirm the impact of FUS_H517D mutations on cell viability of Tet-on LMNs, we used independent cell lines and compared cell viability to control cell lines (Supplementary Figure 2). As a result, we obtained consistent results from two independent clones, showing lower viability of FUS_H517D LMNs (Supplementary Figure 2). We then conducted Sholl analysis on FUS_H517D Tet-on LMNs at DIV7 and DIV11 to assess neurite degeneration. Sholl analysis uses concentric circles to measure neurites intersections at fixed distances from the cell body (Sholl and Uttley, 1953; Binley et al., 2014; O’Neill et al., 2015). This analysis evaluates the number of neurites, dendritic branch geometry, and overall branching patterns. No differences were observed in the Sholl analysis between FUS_H517D Tet-on LMNs and control Tet-on LMNs at either time point (Figures 5A,B). To test the impact of oxidative stress on FUS_H517D Tet-on LMNs’ neurite morphology, we removed antioxidants from the culture media to impose oxidative stress without causing significant cellular damage. Interestingly, the results of Sholl analysis revealed that dendritic complexity and branching patterns were significantly decreased in FUS_H517D Tet-on LMNs compared with controls at DIV11 (Figure 5D). However, when we focused on the result of Sholl analysis at DIV7 under oxidative stress, we could not find a significant difference between FUS_H517D Tet-on LMNs and control Tet-on LMNs (Figure 5C).

Oxidative stress contributes to the differences in neurite arborization observed between FUS_H517D Tet-on LMNs and control cells, suggesting that FUS_H517D Tet-on LMNs are more sensitive to oxidative stress. To further investigate the relationship between oxidative stress and neurite morphology in FUS_H517D Tet-on LMNs, we examined intracellular levels of reactive oxygen species (ROS). ROS were detected using CellROX Green (Thermo Fisher Scientific), a fluorescent dye that binds to DNA and emits bright green fluorescence upon oxidation by ROS. Cells were cultured for 24 h in antioxidant-free (-AO) media, after which ROS levels were assessed. Under -AO conditions, both FUS_H517D Tet-on LMNs and control cells exhibited strong nuclear CellROX Green fluorescence, with no significant difference between the two groups (Figures 5E,F). In contrast, under conventional culture conditions, FUS_H517D Tet-on LMNs showed a significantly higher proportion of CellROX Green–positive nuclei than controls (Figures 5E,F), indicating that these cells experience elevated ROS levels even under conventional conditions.

We also examined the subcellular localization of the FUS protein in FUS_H517D Tet-on LMNs. As expected, FUS aggregates and diffuse cytoplasmic FUS were detected in FUS_H517D Tet-on LMNs but barely in controls, both at DIV7 and DIV35 (Figures 6A–C). To assess the extracellular localization of FUS in cultured cells, we classified FUS distribution in neurons into three distinct categories (Figure 6B): (1) nuclear localization of FUS (representative image shown in Figure 6Ba), (2) extranuclear (cytosolic or aggregated) localization of FUS (representative images shown in Figures 6Bb,c), and (3) condensed nuclei lacking detectable FUS localization (representative image shown in Figure 6Bd). Quantitative analysis demonstrated a significant increase in the proportion of cells exhibiting cytosolic FUS localization in the FUS_1_4 at DIV35 compared to the control (Figure 6C). Notably, our results demonstrated that even in antioxidant-containing media, prolonged culture led to neurite swelling and degeneration in FUS_H517D Tet-on LMNs by DIV35 (Figures 6A–C).

Taken together, these findings, along with data in Figures 2–6, confirm that FUS_H517D Tet-on LMNs exhibit cellular phenotypes identical to those in the ALS model previously reported by Ichiyanagi et al., 2016. Notably, the Tet-on NEUROG2/ISL1/LHX3 iPSCs used for rapid differentiation into a pure neuronal culture allowed us to rule out contamination from other cell types and batch effects (Figures 2A,B).

### 3.3 The microfluidic device is useful for detecting the axonal growth defect in FUS_H517D Tet-on LMNs.

Our results in Figure 5 demonstrate that neurite outgrowth arrest and/or shrinkage, leading to reduced neurite complexity, occurs in FUS_H517D Tet-on LMNs by day 11 post-differentiation under oxidative stress. However, Sholl’s analysis does not specifically reveal axon-specific growth arrest or degeneration, as it primarily focuses on dendritic structure. Since axonal dysfunction and degeneration are implicated in ALS pathogenesis (Coleman, 2022), it is crucial to investigate the axonal phenotype of FUS_H517D Tet-on LMNs.

To address this issue, we aimed to develop analytical methods to assess axonal phenotypes of Tet-on LMNs under oxidative stress. We measured and quantified axonal length in the axonal growth compartment for FUS_H517D Tet-on LMNs using the microfluidic device. In our experiments, both control and FUS_H517D Tet-on LMNs extended axons over 1,000 μm in length within 7 days, with micro-slits nearly fully occupied (Figure 7A). We measured axonal length extending into the axonal compartment (a.c) passing through 1,000 μm long micro-slits (m.s) to compare axonal growth competency. Interestingly, the axonal length of FUS_H517D Tet-on LMNs that extended into the axonal compartment (a.c.) was significantly shorter than that of control Tet-on LMNs at both DIV7 and DIV11 (Figures 7B,C). However, the number of axons reaching the axonal compartment was comparable between FUS_H517D and control Tet-on LMNs at these time points (Figure 7E), suggesting that axonal outgrowth in FUS_H517D Tet-on LMNs was slowed down but not actively retracted at this stage under the oxidative stress. By DIV14, the length of axons detected in the axonal compartment was markedly shortened in FUS_H517D Tet-on LMNs, indicating accelerated axonal retraction (Figure 7D). Consistently, the number of axons of FUS_H517D Tet-on LMNs reaching the axonal compartment was significantly decreased (Figure 7E). Compared to the Sholl analysis results (Figures 5A–D), the axonal phenotype manifests earlier than the general neurite phenotype in FUS_H517D Tet-on LMNs. These findings demonstrate that the microfluidic device is more sensitive to detecting axonal growth defects or arrests in FUS_H517D Tet-on LMNs.

### 3.4 The microfluidic device is compatible with detecting abnormal axonal transport of mitochondria in FUS_H517D Tet-on LMNs

**FIGURE 8:**
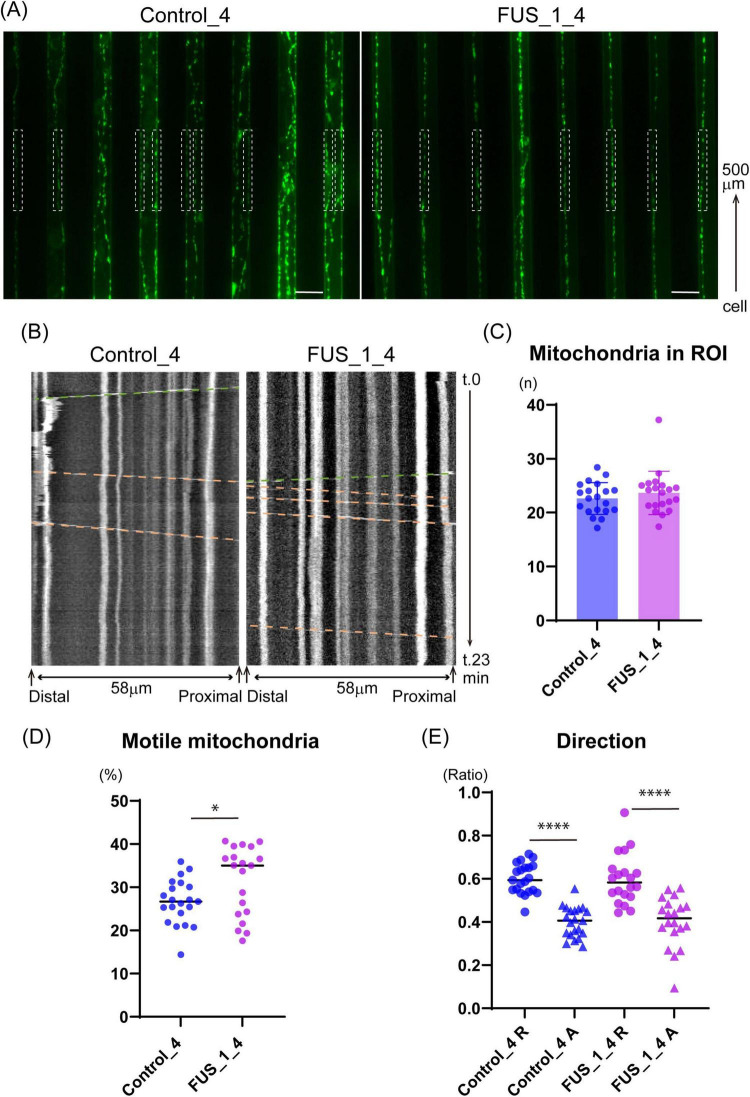
FUS_H517D Tet-on LMNs show altered mitochondria trafficking. **(A)** Representative images that captured micro-slits in the device. MitoTracker-positive mitochondria (green) were detected in axons in the micro-slits. White dash boxes shown in the pictures indicate the ROI used for Kymograph preparation and analysis. Each ROI was set along with a micro-slit 500 μm away from the cellular compartment. 58 μm long single axons and axon bundles less than 1.6 μm wide were analyzed. **(B)** Representative Kymographs are shown. The horizontal axis indicates the orientation of the axon; the vertical axis indicates the time course. Green dash lines demonstrate the trajectory of anterograde transport, whereas orange dash lines demonstrate the trajectory of retrograde transport. **(C)** The average number of mitochondria in each observation field is shown. The average number of mitochondria in ROI was comparable between Control_4 and FUS_H517D Tet-on LMNs. [Median; Control_4, 22.75 (*n* = 21) and FUS_H517D, 22.94(*n* = 20)]. **(D)** The percentage of motile mitochondria is shown. [Median; Control_4, 26.71 (*n* = 21) and FUS_H517D, 35.03 (*n* = 20)]. Mann-Whitney Test. *p < 0.05. **(E)** Direction of transport is analyzed. The ratio of retrograde transport shares a large part of trafficking in both Control_4 and FUS_H517D Tet-on LMNs. The difference between the groups was assessed by the Kruskal-Wallis Test, followed by Dunn’s multiple comparisons test. ****p < 0.0001.

To further explore the advantages of our device for analyzing FUS_H517D Tet-on LMNs, we quantified mitochondrial axonal transport in FUS_H517D Tet-on LMNs. Dysregulation of axonal transport is implicated in neurodegenerative diseases, including ALS (De Vos and Hafezparast, 2017; Bilsland et al., 2010). Axonal transport is essential for delivering organelles and vesicles carrying proteins and mRNA to the distal parts of the neuron, which supports axonal growth and maintenance. The transport of cargo between the cell body and the distal axon depends on motor proteins that require ATP (Hirokawa et al., 2010). Given that axonal energy is mainly supplied by local mitochondria and enhanced mitochondrial trafficking supports axonal regeneration (Zhou et al., 2016), we hypothesized that alterations in mitochondrial trafficking in the axons of FUS_H517D Tet-on LMNs could contribute to axon degeneration. To test this hypothesis, we conducted real-time imaging of mitochondrial transport in axons using our microfluidic device. Cells were cultured in normal growth media since maintaining cell culture conditions for real-time imaging under oxidative stress (without antioxidants) proved challenging. We cultured FUS_H517D Tet-on LMNs in the microfluidic device for 13–15 days and observed mitochondrial transport within a region of interest (ROI) for 23 min. The ROI, located 500 μm from the cellular compartment and measuring 58 μm in length, was positioned along a micro-slit (Figure 8A). Axonal bundles thicker than 1.6 μm in diameter were excluded from analysis.

Previous studies have shown that the ratio of motile mitochondria and transport direction can vary in axonal regions *in vitro* (Abo-Rady et al., 2020). To ensure consistency, we positioned the ROI in the same location within the microfluidic device across experiments. The device, placed on a cover glass, allowed clear observation of axonal mitochondria stained with MitoTracker Green within the micro-slits (Figure 8A). First, we confirmed that the number of mitochondria in the ROI was comparable between FUS_H517D and control Tet-on LMNs (Figure 8B), ensuring equivalent experimental conditions. Next, we quantified the percentage of motile mitochondria in axons using kymograph-based trajectory analysis (Figure 8B). Consistent with previous findings that 20–30% of mitochondria are motile in axons (Chen and Sheng, 2013; Lin and Sheng, 2015), we observed that approximately 30% of mitochondria were motile in both control and FUS_H517D Tet-on LMNs (Figure 8C). Interestingly, the percentage of motile mitochondria in FUS_H517D Tet-on LMNs was significantly higher than in control cells [Median: Control, 26.48% (*n* = 21); FUS_H517D, 35.03% (*n* = 20)] (Figure 8D). We then examined the direction of mitochondrial transport. In both cell lines, retrograde transport represented a larger proportion of the total transport (Figure 8E). Although previous research indicated increased retrograde mitochondrial transport in SOD1 mutant-expressing neurons compared to controls (De Vos et al., 2007), we did not observe a significant difference in retrograde transport between FUS_H517D Tet-on LMNs and control cells (Figure 8D).

To investigate whether mitochondrial defects contribute to abnormal trafficking, we assessed mitochondrial membrane potential in FUS_H517D Tet-on LMNs using the MT-1 fluorescent dye, a membrane potential indicator (Inoue et al., 2022). Treatment with CCCP (carbonyl cyanide m-chlorophenyl hydrazone), a mitochondrial uncoupler reagent, significantly reduced MT-1 signal intensity in both control and FUS_H517D Tet-on LMNs, confirming MT-1’s responsiveness to changes in membrane potential (Supplementary Figures 4A,B). Treatment with valinomycin, a potassium-selective ionophore, caused a slight increase in MT-1 intensity, but this change was not statistically significant in either cell line (Supplementary Figures 4A,B). Notably, we found no significant difference in MT-1 signal intensity between control and FUS_H517D Tet-on LMNs, indicating that mitochondrial membrane potential is preserved in FUS_H517D Tet-on LMNs at this stage (Supplementary Figures 4A,B).

## 4 Discussion

In this study, we developed a microfluidic device designed for culturing LMNs derived from human iPSCs (Figures 1A–D) and evaluated its effectiveness in uncovering axonal phenotypes. First, we investigated the cellular phenotypes of FUS_H517D Tet-on LMNs in detail (Figures 2–6). Given that FUS_H517D Tet-on LMNs are induced by the transient expression of transcription factors, we considered the potential impact of this forced expression on cell viability and differentiation efficiency. As anticipated, some cell death occurred during transcription factor induction; however, the viability of FUS_H517D Tet-on LMNs at DIV7 was comparable to that of control Tet-on LMNs (Figure 4A; Supplementary Figure 2). In contrast, at DIV14 and DIV21, FUS_H517D Tet-on LMNs displayed significantly lower viability than control Tet-on LMNs (Figure 4B; Supplementary Figure 2), suggesting that these LMNs are prone to die after differentiation.

Our data indicates that H517D mutations in the *FUS* gene impacted LMN survival, with degeneration being present yet mild under conventional media conditions (Figures 4–6). The neurite complexity of FUS_H517D Tet-on LMNs, as shown by Sholl analysis at DIV7 and DIV11, was similar to that of control cells (Figures 5A,B). However, when cultured without antioxidants, the neurite complexity of FUS_H517D Tet-on LMNs significantly declined at DIV11, indicating that FUS_H517D Tet-on LMNs were more susceptible to oxidative stress (Figure 5C). Consistently, cellular ROS levels rise in FUS_H517D Tet-on LMNs even in conventional media, suggesting that they require higher oxidative stress management than control cells (Figure 5D,E). Notably, axonal growth arrest in FUS_H517D Tet-on LMNs under oxidative stress conditions was detectable using our device as early as DIV7 (Figure 7). This alteration in neuronal morphology was the earliest observable phenotype in FUS_H517D Tet-on LMNs and would have been undetectable without the use of this device. Thus, our microfluidic device may serve as a valuable tool for identifying axon-specific phenotypes at the early stages of neuronal degeneration. The arrest and retraction of neurite and axon growth are the cellular phenotypes frequently reported in cellular models of neurodegenerative diseases, and this phenotype is one of the indicators for the efficacy and toxicity evaluation of new drugs (Fujimori et al., 2018). Although we haven’t tested the efficacy of a candidate ALS drug on axonal degeneration phenotypes in FUS_H517D Tet-on LMNs using our device, we have preliminary results showing that we can detect the effect of the addition of antioxidants in media on the axonal growth and prevention of axonal degeneration (Supplementary Figure 5). Therefore, our device, which can sensitively sense and easily quantify neuronal axonal phenotypes, can potentially increase the efficiency of evaluating the efficacy and toxicity of new drugs.

It is known that differentiating human iPSCs into neurons via dual SMAD inhibition is a lengthy and complex process (Chambers et al., 2009; Surmacz et al., 2012), often resulting in batch-to-batch variability in differentiation efficiency. LMNs differentiated from ALS patients’ iPSCs using this method are commonly utilized for ALS drug screening and evaluation. However, to reliably assess drug candidates for ALS, verifying and controlling the cellular characteristics and quality of each batch of the ALS cellular model is essential. While this step is critical, it is challenging to maintain consistent control over all aspects of differentiation. In this regard, our ALS cellular model, FUS_H517D Tet-on LMNs, offers distinct advantages for ALS drug screening. The differentiation efficiency of FUS_H517D Tet-on LMNs exceeds 95% (Figures 1A,B), and the protocol is both reproducible and straightforward. Furthermore, these cells reliably exhibit ALS-relevant phenotypes previously reported (Ichiyanagi et al., 2016), including accelerated cell death post-differentiation (Figures 3A,B), neurite degeneration (Figures 4A–D, Figure 7), and cytoplasmic mislocalization of the FUS protein (Figure 5). In addition to them, we show that FUS_H517D Tet-on LMNs reveal abnormal mitochondria trafficking in axons (Figures 8A–E).

Axonal trafficking is a key area of research for understanding the pathogenesis of ALS (Millecamps and Julien, 2013; De Vos and Hafezparast, 2017; Münch et al., 2005). The transport of various organelles—including mitochondria, endosomes carrying trophic signaling receptors, and autophagosomes/lysosomes—has been reported to be disrupted in ALS models (Bilsland et al., 2010; Magrané et al., 2014; Liu et al., 2021). Among these, the molecular mechanisms underlying impaired mitochondrial trafficking and its contribution to neuronal degeneration have been extensively studied and widely recognized. For example, iPSC-derived LMNs carrying TDP-43 mutations exhibit reduced mitochondrial motility, which has been linked to the downregulation of dynactin (Dafinca et al., 2024). Similarly, LMNs from SOD1_G93A transgenic mice show impaired dynein-mediated retrograde transport (Kieran et al., 2005). Mitochondria play a crucial role in neurons by providing ATP through oxidative phosphorylation, which is essential for meeting the high metabolic demands of these cells. In particular, mitochondria anchor to microtubules in specific axonal regions where energy demand is higher, such as presynaptic boutons and axon terminals. These stationary mitochondria provide ATP and buffer calcium ions within the axon (Sheng, 2014). Our results on mitochondrial trafficking indicate that stationary mitochondria are reduced in the axons of FUS_H517D Tet-on LMNs, suggesting that ATP supply and calcium buffering may be insufficient in these neurons. Interestingly, previous research has shown that FUS interacts with the mitochondrial anchor protein Syntaphilin, and mutations in FUS can disrupt this interaction, resulting in increased mitochondrial trafficking (Salam et al., 2021). Although the specific interaction between FUS_H517D and Syntaphilin remains unanalyzed, it is worthwhile to investigate further the role of the FUS in regulating Syntaphilin and whether FUS_H517D affects the Syntaphilin function.

In recent years, animal testing has been scaled back, with a shift toward alternative methods using human cells differentiated from iPSCs and other sources (Kimura et al., 2024; Ito et al., 2024). Microfluidic devices have become valuable tools for controlling cell culture environments and are increasingly integrated to developing alternative research methods. Our team has developed various microfluidic devices focusing on neuronal analysis (Yokoyama et al., 2019; Otomo et al., 2022). In this study, we introduced a neuron-compartmentalized cell culture device and demonstrated its utility in analyzing axonal phenotypes. Our device allows for effective monitoring of axonal length (Figures 7A–D) and axonal trafficking (Figures 8A–E). Beyond the experiments presented here, our device is possibly helpful for a range of additional applications: 1. Compartment-Specific Sample Collection: By regulating neuronal polarity, our device facilitates the isolation of axonal samples without contamination from other compartments. This setup allows for the analysis of axon-specific mRNA trafficking to understand ALS pathogenesis due to disrupted mRNA trafficking in diseased axons (Alami et al., 2014; Ishiguro and Ishihama, 2023). 2. Co-Culture Experiments: Given that ALS research widely recognizes the non-cell-autonomous mechanism of neuronal degeneration (Van Harten et al., 2021), our device is suitable for exploring ALS cellular models in co-culture with glial cells, such as astrocytes and microglia. This model could provide new insights into ALS by observing how glial cells impact axonal phenotypes within a controlled neuronal environment. 3. Development of Neuromuscular Junctions: The synapse between LMNs and skeletal muscle is a key target in ALS research. However, conventional cell culture systems do not allow for independent stimulation or inhibition of LMN activity, making it difficult to assess synaptic structures or functionality. With our device, we can potentially culture LMNs and muscle cells within specific compartments, enabling compartment-dependent stimulation or inhibition to facilitate functional analysis of neuromuscular junctions.

This study highlights the advantages of using iPSC-derived LMNs and a microfluidic device; however, several limitations should be acknowledged. First, we focused exclusively on a single FUS mutation, FUS_H517D, and demonstrated its association with accelerated axonal phenotypes. To generalize our findings, it is necessary to investigate whether other FUS mutations similarly lead to axonal degeneration in LMNs. Second, our experiments were conducted entirely *in vitro*. As such, we cannot fully capture the axonal phenotypes of FUS_H517D *in vivo*, where the conditions and microenvironment affecting axonal growth are more complex and physiologically relevant. Third, we examined the phenotype of Tet-on FUS_H517D LMNs only during early neuronal development. Consequently, we were unable to assess the phenotypes of mature neurons. Since achieving stable neuronal maturation in Tet-on FUS_H517D LMNs remains technically challenging, further optimization of our culture methods and conditions is required to address this limitation.

Microfluidic devices have seen increased use in neuroscience research, with demand steadily growing in the field. Continued research utilizing devices specifically designed for culturing human iPSC-derived LMNs will enhance our understanding of the degeneration mechanisms in human ALS model cells. Such advancements are expected to play a significant role in evaluating and selecting candidate drugs for ALS treatment.

## Data Availability

The original contributions presented in the study are included in the article/Supplementary material, further inquiries can be directed to the corresponding authors.

## Footnotes

1
https://xonamicrofluidics.com
