# Supplementary material for: Investigation of early axonal phenotypes in an iPSC-derived ALS cellular model using a microfluidic device
Source: Front Cell Neurosci. 2025 Jul 24;19:1590732. doi: 10.3389/fncel.2025.1590732 (PMC12328293; doi:10.3389/fncel.2025.1590732)
Supplement: Supplementary file 1 [file Data_Sheet_1.pdf]

Supplementary file

Supplementary figure legends

Supplementary Figure 1. The illustration shows the procedure for inducing FUS_H517D Tet-on LMNs. NEUROG2, ISL1, and LHX3 are expressed under the control of the TRE promoter. iPSCs were dissociated into a single cell and plated on poly-D-lysine and laminin-coated cells. The cells were treated with doxycycline in differentiation media. After differentiation, the cells were used for analysis.

Supplementary Figure 2. The cell viability of FUS_H517D Tet-on LMNs from two independent iPS clones was analyzed using PrestBlue reagent. To examine the consistency of the impact of FUS_H517D mutations on cell viability, we conducted cell viability assays of FUS_H517D Tet-on LMNs and control LMNs at DIV14 and DIV21. The results show a ratio of cell viability at DIV14 and DIV21 to that at DIV7. A two-way ANOVA was conducted to determine whether genotype and culture duration have an effect on cell viability. The results showed statistically significant variances in both factors (geneotype and time point). However, no significant differences were detected among the groups by *a Bonferroni test.*

Supplementary Figure 3. Representative binary images used in Sholl analysis are shown. The immunofluorescent images for NF200 captured at each time point and condition were converted to binary images. Then, each cell mask was prepared and used for Sholl analysis. The scale bar indicates 50μm.

Supplementary Figure 4. (A) Conrtol_4 and FUS_H517D Tet-on LMNs at DIV14 were treated with DMSO, Valinomycin, and CCCP for 24h and were stained for MT-1 regent. Red signals represent MT-1 staining. The scale bars indicate 20μm. (B) MT-1 signal intensity was shown as the average signal intensity of a single cell. The Kruskal-Wallis test and multiple comparison tests were used to assess the differences between the groups. * $p<0.05$ ** $p<0.01$

Supplementary Figure 5. The effect of the addition of antioxidants on axonal growth and morphology. In oxidative stress conditions, after 3 days of FUS_1_4 culture in the device with conventional media containing antioxidants, the culture media were replaced with antioxidant-free media (-AO), and the cells were continuously maintained by -AO until DIV11. In the rescue conditions (+AO), to investigate the effect of antioxidants in the media on axonal growth and morphology, we replaced -AO media with conventional media containing antioxidants at DIV4, and continued culture until DIV11. The scale bars indicate 0.1mm.

Supplementary method

2.1 Comparison of mitochondrial membrane potential.

After 24h of DMSO (0.02%), Valinomycin (20μM), or CCCP (50μM) treatment, MT-1 assay was conducted. Cells were incubated with media containing MT-1 dye (Dojin) diluted (1:1000) for 30 minutes. Then, cells were washed with fresh media, fixed with 4% PFA, and imaged. Signal intensity corresponding to MT-1 staining in images was measured and calculated. MT-1 signal intensity was shown as the average signal intensity of a single cell (n=16-19).

2.2 Examination of the effect of antioxidants on axonal growth and morphology.

In oxidative stress conditions, after 3 days of FUS_1_4 culture in the device with conventional media containing antioxidants, the culture media were replaced with antioxidant-free media (-AO), and the cells were continuously maintained by -AO until DIV11. In the rescue conditions, to investigate the effect of antioxidants in the media on axonal growth and morphology, we replaced -AO media with conventional media containing antioxidants (+AO) at DIV4, and continued culture until DIV11. Half the volume of the medium in the media port (120μl) was replaced every two days. Representative preliminary results were shown.

Supplementary Table 1. Primary antibodies used in this study were listed.

Primary antibody	Name	Cat.No	Industry	Host
#1	NF200	N4142	SIGMA	Rabbit
#2	HB9	sc-515769	Santa Cruz	Mouse
#3	TUBB3	801213	BioLegend	Mouse
#4	ChAT4B1	AB_528122	DSHB	Mouse

Supplementary Table 2. Primer sequences used in this study were listed.

Target gene	Forword	Reverse
CHAT	5'-ACATGATTGAGCGCTGCATC-3'	5'-ACTTGTCGTACCAGCGATTG-3'
MNX1	5'-AGCACCAGTTCAAGCTCAAC -3'	5'-TGGCCTTTTTGCTGCGTTTC-3'
NANOG	5'-GCTTGCCTTGCTTTGAAGCA-3'	5'-TTCTTGACCGGACCTTGTC-3'
GAPDH	5'-GATGAGAAGTATGACAACAGCC-3'	5'-AGTCCTTCCACGATACCAAAGT-3'
LHX3	5'-TGCAGGTTTGGTTCCAGAACCG-3'	5'-GCCAGGCCTCCATGCTCCAGGG-3'
TUBB3	5'-ATTTCATCTTTGGTCAGAGTGG-3'	5'-TGCAGGCAGTCGCAGTTTTCAC-3'

Supplementary movie 1. Representative movie of mitochondria trafficking in Control_4 Tet-on LMNs. Its screen size is 8.28 μm in width and 82.8 μm in height.

Supplementary movie 2. Representative movie of mitochondria trafficking in FUS_H517D Tet-on LMNs. Its screen size is 8.28 μm in width and 82.8 μm in height.
