# Supplementary figures and images for: Investigation of early axonal phenotypes in an iPSC-derived ALS cellular model using a microfluidic device
Source: Front Cell Neurosci. 2025 Jul 24;19:1590732. doi: 10.3389/fncel.2025.1590732 (PMC12328293; doi:10.3389/fncel.2025.1590732)

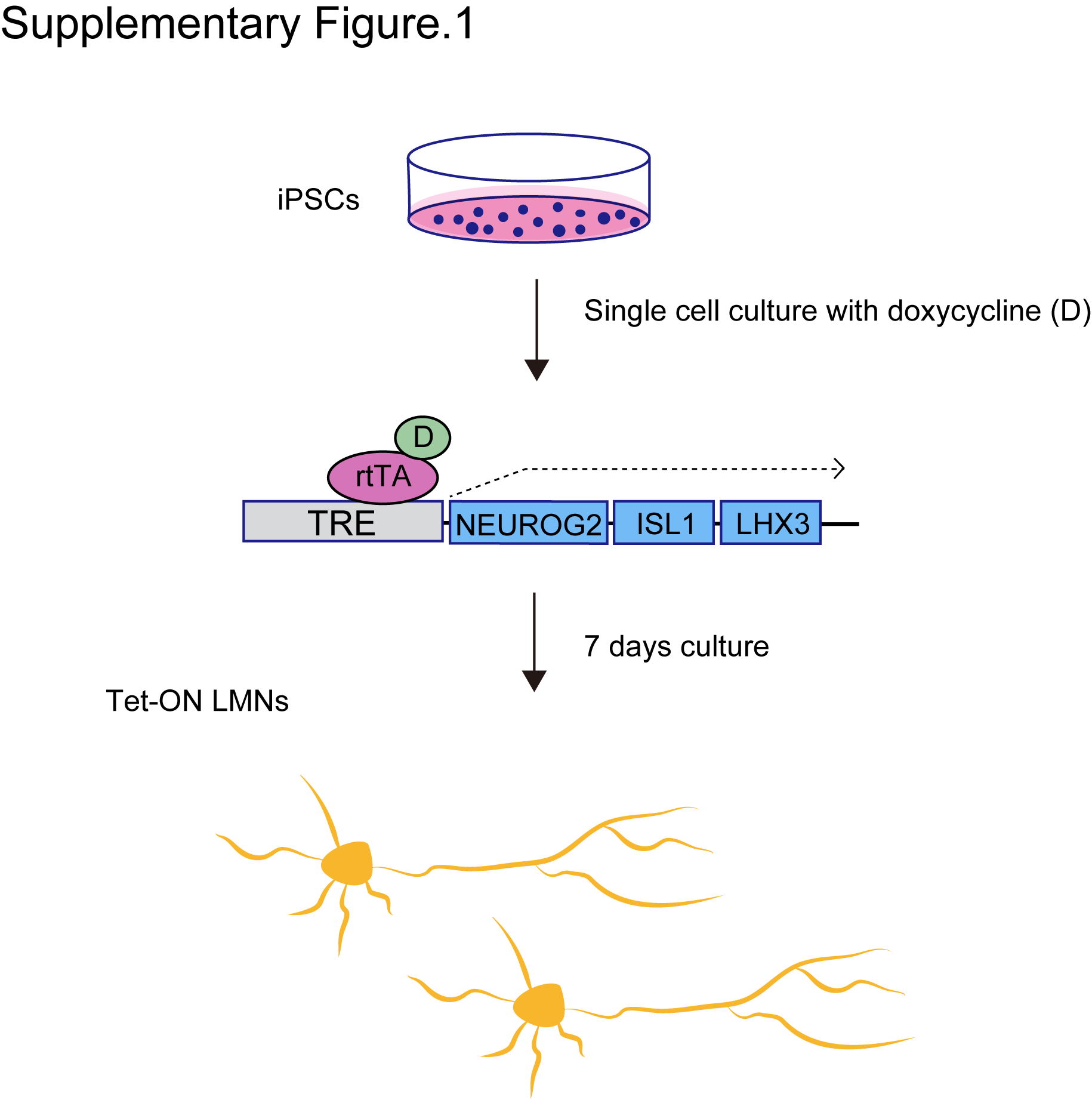

Supplement: Supplementary file 4 [file Image_1.tif]

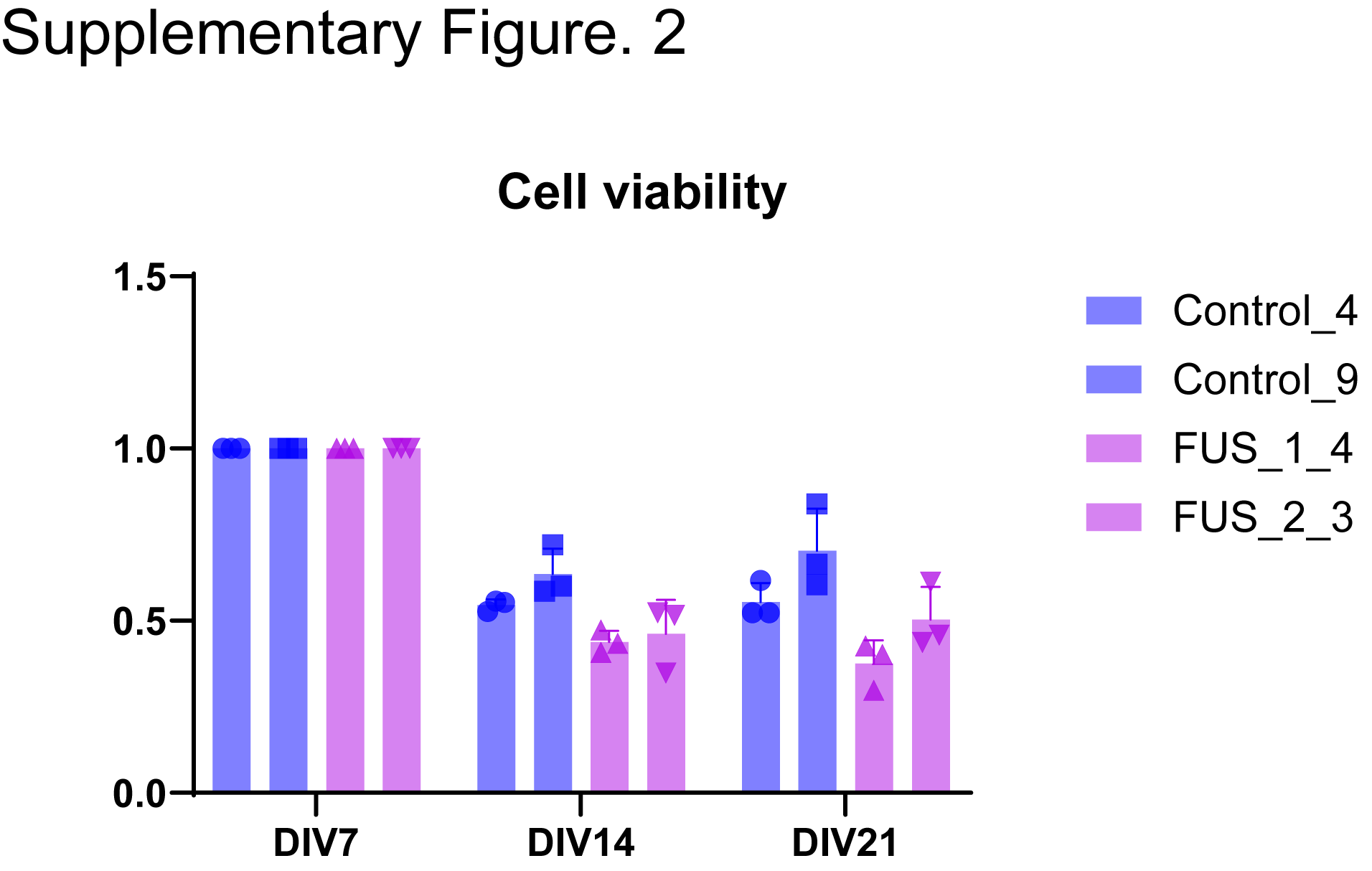

Supplement: Supplementary file 5 [file Image_2.tif]

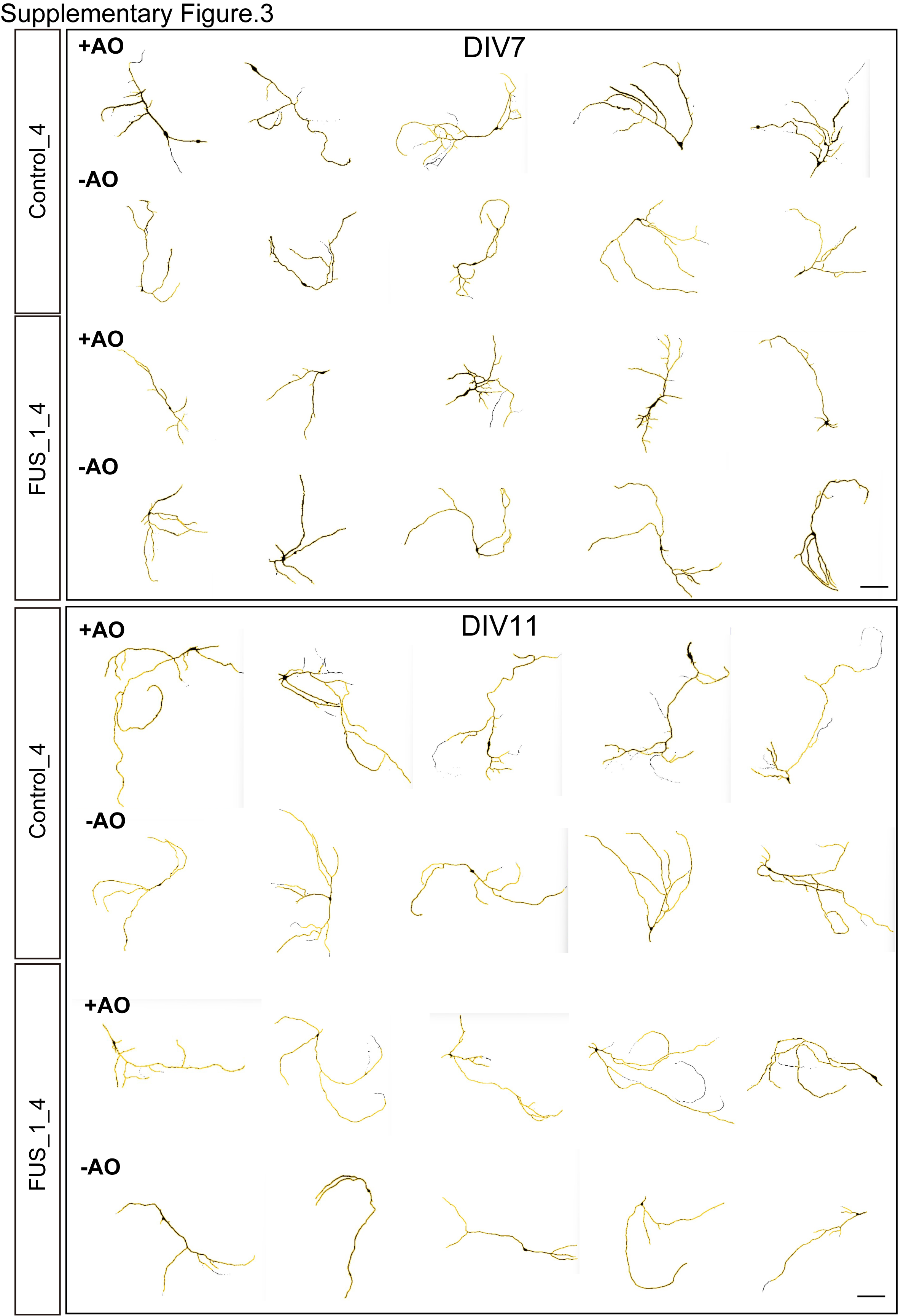

Supplement: Supplementary file 6 [file Image_3.tif]

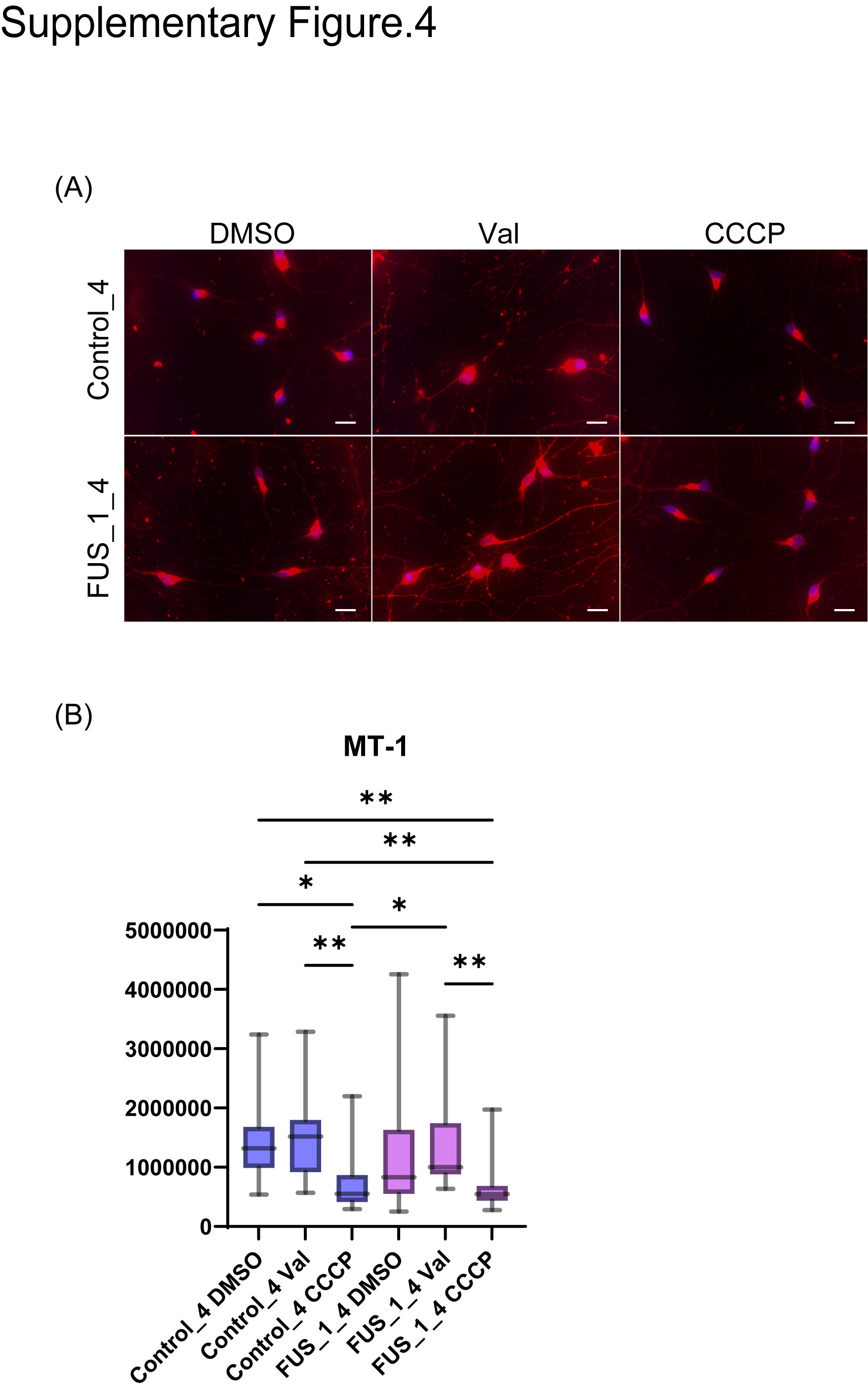

Supplement: Supplementary file 7 [file Image_4.tif]

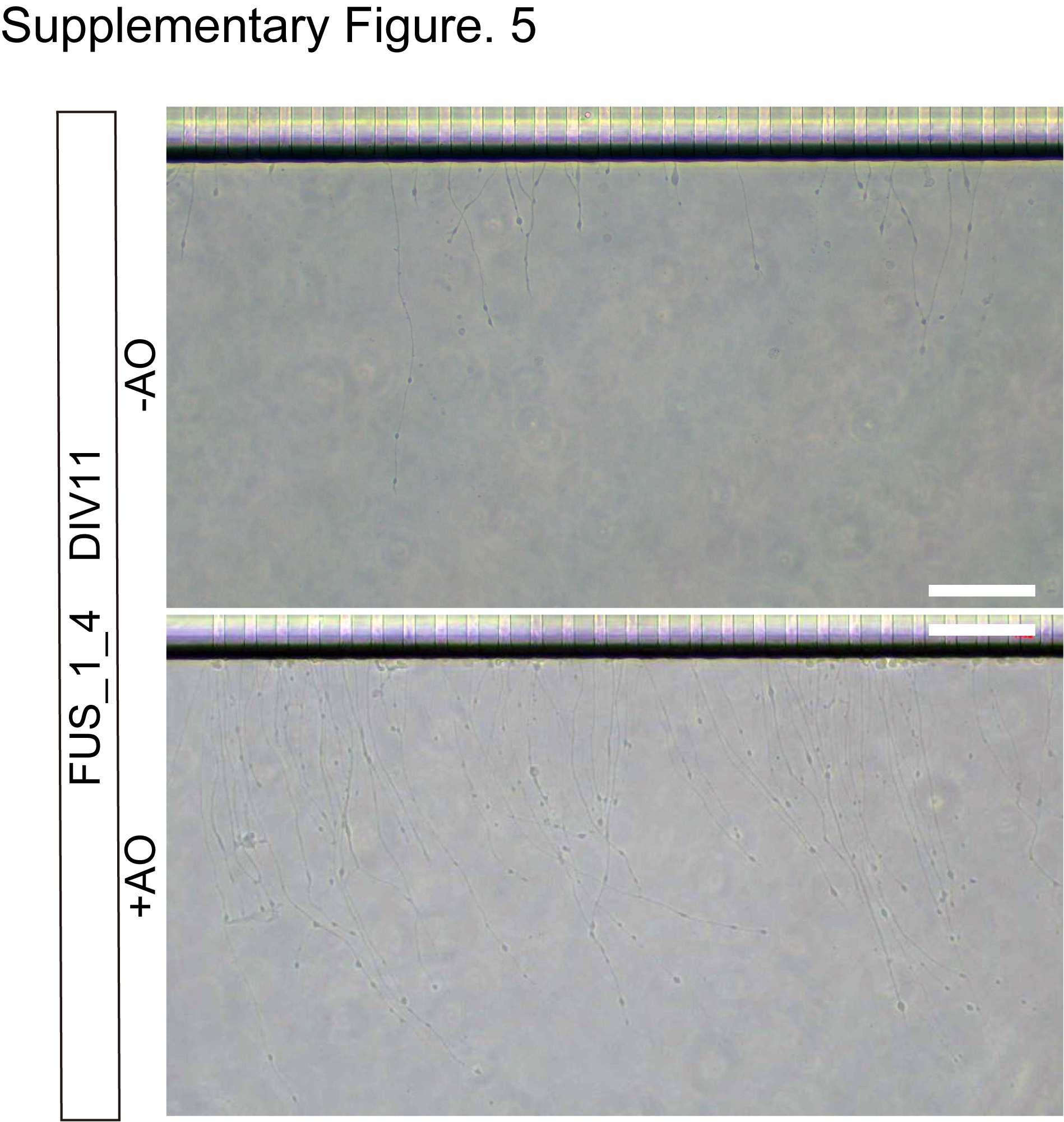

Supplement: Supplementary file 8 [file Image_5.tif]
